# Phase-specific mortality risk of serum lactate thresholds in very low birth weight infants with late-onset sepsis: a retrospective cohort study

**DOI:** 10.3389/fmed.2025.1662406

**Published:** 2025-09-04

**Authors:** Yu Lun, Zuming Yang, Yanhong Li

**Affiliations:** ^1^Department of Nephrology and Immunology, Children’s Hospital of Soochow University, Suzhou, China; ^2^Department of Neonatal Intensive Care Unit, Suzhou Municipal Hospital, Suzhou, China; ^3^Institute of Pediatric Research, Children’s Hospital of Soochow University, Suzhou, China

**Keywords:** late-onset sepsis, very low birth weight infants, lactate, threshold effect, mortality late-onset sepsis, mortality

## Abstract

**Background:**

Late-onset sepsis (LOS) in very low birth weight (VLBW) infants confers substantial mortality risk. While lactate monitoring is standard, validated prognostic thresholds for mortality remain unestablished in this population.

**Methods:**

This retrospective cohort study (2014–2024) analyzed 596 VLBW infants (≤32 weeks; <1,500 g) with LOS at a tertiary NICU. Multivariable regression and piecewise linear modeling identified lactate-mortality thresholds, adjusting for gestational age, respiratory failure severity, vasopressor requirement, and multiorgan dysfunction. Bootstrap validation (1,000 iterations) assessed threshold stability.

**Results:**

Mortality occurred in 21% (125/596). Nonlinear analysis revealed critical inflection points at 2.2 mmol/L (95%CI: 1.9–2.5) and 4.0 mmol/L (95%CI: 3.7–4.3) (*p* < 0.001). Lactate ≤ 2.2 mmol/L demonstrated no mortality association (adjusted odds ratio = 1.84, 95%CI: 0.64–5.34; *p* = 0.260). Within the 2.2–4.0 mmol/L transition zone, each 1 mmol/L increment conferred a 7.0-fold mortality risk (aOR = 7.0, 95%CI: 2.13–22.78; *p* < 0.001). Beyond 4.0 mmol/L, the relationship attenuated (aOR = 0.90, 95%CI: 0.52–1.43; *p* = 0.568). Subgroup analyses indicated amplified risk among epinephrine-exposed infants (aOR = 3.40 vs. 1.78; *P*_interaction_ = 0.094) and those with moderate-to-severe respiratory failure.

**Conclusion:**

Lactate reveals phase-specific mortality associations in VLBW infants with LOS. The 7.0-fold mortality risk increase per mmol/L in the 2.2–4.0 mmol/L interval suggests potential metabolic resuscitation targets for precision monitoring in sepsis management.

## 1 Introduction

Late-onset sepsis (LOS) in very low birth weight (VLBW) infants remains a critical contributor to neonatal mortality, responsible for 16–25% of NICU deaths despite therapeutic advances (1, 2). The convergence of rising multidrug-resistant infections and prolonged hospitalizations highlights an urgent clinical priority: bedside-capable tools for rapid mortality risk stratification at symptom onset. While existing multiparameter systems provide valuable prognostic insights, no single pathophysiological anchored biomarker currently enables immediate risk triage in the critical 6-h window–a pivotal gap for guiding early targeted interventions in this vulnerable population (3).

Serum lactate – an easily and simply accessible laboratory parameter during hypoperfusion – is an established prognostic predictor in adult and pediatric sepsis, with levels exceeding 4 mmol/L strongly correlating with microcirculatory collapse and poorer prognosis (4–6). In neonates, however, hyperlactatemia manifests fundamentally distinct pathobiology due to developmental immaturity: constrained hepatic clearance from deficient hepatocyte HAGH enzyme activity, coupled with stress-induced mitochondrial dysfunction that shifts metabolism toward accelerated glycolysis (7, 8). When applied to late-onset sepsis (LOS) in VLBW infants, these physiological singularities generate critical knowledge gaps. Conventional linear prognostic models fail to capture the nonlinear mortality dynamics observed in this population (9). Furthermore, prognostic effect modifiers including oxygenation impairment severity and vasoactive agent burden may significantly modulate lactate’s risk stratification utility, yet subgroup-specific validation remains absent (10).

To address these gaps, we conducted a retrospective cohort study of very low birth weight (VLBW) infants with late-onset sepsis (LOS). Using segmented regression with comprehensive adjustment for illness severity, vasopressor exposure, and key confounders, we quantified lactate’s mortality relationship across clinical phenotypes, establishing precision risk stratification thresholds for this vulnerable population.

## 2 Materials and methods

### 2.1 Study population and design

This study was designed as a retrospective analysis of data collected from neonates diagnosed with late–onset sepsis who were admitted to Suzhou Municipal Hospital, from January 1, 2014 to December 31, 2024.

Inclusion criteria were as follows: (a) gestational age of ≤ 32 weeks and (b) birth weight ≤ 1,250 g.

Exclusion criteria included: (a) infants with missing information and (b) infants whose guardians abandoned or withdrew them from treatment within 7 days of the onset of sepsis.

**Figure 1:**
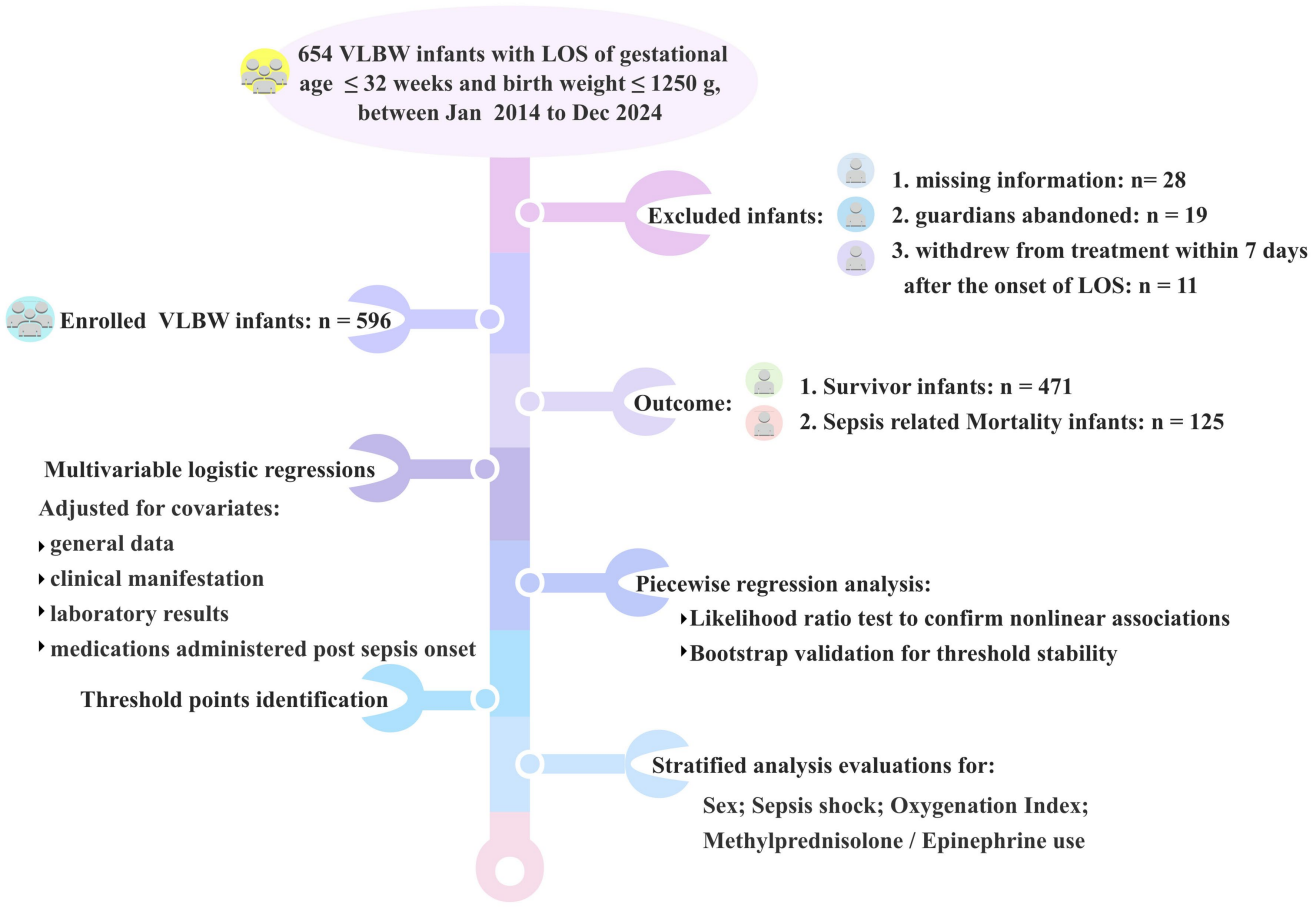
Study flowchart: patient selection and analytical framework.

A flowchart was provided to illustrate the detailed process (Figure 1).

### 2.2 Measurements and definitions

LOS was defined as (1) sepsis occurring after 72 h of life (1), (2) blood culture was drawn, and empirical antimicrobial therapy was initiated at the time of evaluation, continuing for a minimum of 5 days or until the patient’s demise (13) (11).

The operational definition of sepsis episodes was established through standardized multiorgan dysfunction criteria requiring concurrent documentation of ≥ 2 systemic manifestations across distinct organ systems, including: (1) thermal dysregulation: hypothermia (core temperature < 36.0 °C) or hyperthermia (core temperature > 38.0 °C); (2) neurological compromise: hypotonia or lethargy; (3) cardiovascular instability: tachycardia (> 180 bpm) or bradycardia (< 85 bpm), poor peripheral perfusion, characterized by pale, cold, and mottled skin, and concurrent perfusion deficits quantified by capillary refill time > 3 s; (4) respiratory deterioration: increased frequency of apnea or the emergence of new episodes, and an elevated requirement for respiratory support; and (5) gastrointestinal dysfunction: abdominal distension accompanied by decreased feeding tolerance (12).

All laboratory parameters—arterial blood gases, white blood cell counts (WBC), absolute neutrophil counts, platelet counts (PLT), and serum inflammatory biomarkers C–reactive protein (CRP), procalcitonin (PCT)—were analyzed from blood samples collected within 30 min of suspected sepsis onset. Additionally, clinical parameters—including oxygenation index (OI), administration of methylprednisolone or epinephrine, and presence of septic shock—were recorded within 2 h of sepsis onset.

The endpoint event in this study was sepsis–related mortality, which defined as any fatalities occurring within 7 days of a positive blood culture or in the presence of clinical and laboratory evidence of sepsis despite negative blood cultures (13).

### 2.3 Covariate assessment

Clinical characteristics and laboratory results were obtained from the hospital’s electronic medical record system, following a predetermined protocol. The data collection encompassed the following categories: (1) general data, including gestational age, birth weight, sex, and maternal perinatal risk factors; (2) clinical manifestations, such as apneic episodes, lethargy, tachycardia or bradycardia, abdominal distension, and skin perfusion abnormalities indicative of microcirculatory irregularities; (3) laboratory results, comprising routine blood tests (white blood cell counts, absolute neutrophil counts, and platelet counts), biochemical indices (procalcitonin and C–reactive protein), and arterial blood gas analyses (pH values, blood lactate levels, and blood glucose concentration), along with blood culture results (Gram-negative bacteria, Gram-positive bacteria, fungemia, and culture-negative); (4) other factors associated with sepsis, including septic shock, and the oxygenation index (OI), calculated as [(FiO2 × Mean airway pressure × 100) ÷ PaO2]; (5) medications administered post–onset, methylprednisolone (1– 2 mg/kg/day) and epinephrine (0.1– 1 μg/kg/min) (14).

### 2.4 Statistical analysis

Continuous variables were expressed as mean ± standard deviation (SD) or median with interquartile range (IQR; P25–P75) based on normality assessed by Shapiro–Wilk tests. Categorical variables were presented as counts and percentages (%). For between–group comparisons, survivors and non–survivors were analyzed using Wilcoxon rank–sum tests for non–normal continuous variables and *χ*^2^ tests (or Fisher’s exact tests for sparse data) for categorical variables. Group comparisons were performed with chi-square tests followed by Bonferroni correction for multiple testing.

To evaluate the association between initial blood lactate levels and sepsis–related mortality, multivariable logistic regression models were sequentially constructed: Firstly, Model 1 (crude, unadjusted) estimated the univariate effect of lactate. Subsequently, Model 2 adjusted for Birth weight, Gestational age, Sex, Sepsis shock, Methylprednisolone use, and Epinephrine use. Finally, Model 3 fully adjusted for birth weight, gestational age, sex, sepcies of pathogens bacteria, sepsis shock, methylprednisolone use, epinephrine use, tachycardia (>180 bpm), bradycardia (<85 bpm), pH, glucose, white blood cell counts (WBC), absolute neutrophil counts, platelet counts (PLT), procalcitonin (PCT), C-reactive protein (CRP), and oxygenation index (OI). To assess multicollinearity among variables, the variance inflation factor (VIF) was utilized, with a value of VIF ≥ 5 signifying the existence of multicollinearity.

Additionally, a generalized additive model (GAM) was used to identify the nonlinear relationship between lactate levels and sepsis–related mortality. Following visual identification of nonlinearity, a two–piecewise linear regression model (R segmented package) was implemented to quantify threshold effects. The lactate inflection point was determined by maximum likelihood estimation, and its stability was validated via nonparametric bootstrap resampling (1,000 iterations) to derive bias–corrected 95% confidence intervals (CIs).

Prespecified subgroup analyses assessed effect modification of the lactate-mortality association by sex, septic shock status, methylprednisolone use, epinephrine use, and oxygenation impairment severity [categorized by Oxygenation Index (OI): < 8 indicating no-to-mild impairment, ≥ 8 indicating moderate-to-severe impairment] (15, 16). Multiplicative interaction terms (lactate × subgroup) were incorporated into fully adjusted logistic regression models following confirmation of minimal multicollinearity (all variance inflation factors < 3). Statistical interaction was evaluated using likelihood ratio tests comparing nested models with and without interaction terms.

Statistical significance was defined as a *p*-value less than 0.05 in a two-tailed test. Data analyses were performed using R software (version 4.4.0) and Empower Stats software (version 6.0).^1^

## 3 Results

### 3.1 Study population and baseline characteristics

The retrospective cohort comprised 596 VLBW infants diagnosed with late–onset sepsis (culture–positive or clinically confirmed), including 125 sepsis–related deaths (21.0%) and 471 survivors (79.0%). Non-survivors exhibited significantly lower birth weight than survivors (median 860 g [780, 950] vs. 930 g [830, 1,050]; *p* < 0.001), while gestational age did not differ (median 28.2 weeks [27.6, 28.9] vs. 28.3 weeks [27.5, 29.4]; *p* = 0.398). Birth asphyxia resuscitation indicators showed no significant differences between survivors and non-survivors: 1-min Apgar scores (median 8 [IQR 7–8] vs. 8 (7, 8); *p* = 0.080), with a similar non-significant trend at 5 min (*p* = 0.137). Antenatal corticosteroids were administered to 95.8% of the cohort, with comparable exposure between survivors (96.4%) and non-survivors (93.6%) (*p* = 0.166). Maternal antenatal complications—including prolonged membrane rupture, gestational hypertension, and diabetes—did not differ significantly between survivors and non-survivors (all *p* > 0.05; Table 1).

**Table 1:** Demographic characteristics of VLBW infants with LOS.

Variables	Total (*n* = 596)	Survivor (*n* = 471)	Non-survivor (*n* = 125)	*p*
Birth weight (g)	910 (820, 1,050)	930 (830, 1,050)	860 (780, 950)	**<0.001**
Gestational age (weeks)	28.3 (27.6, 29.1)	28.3 (27.5, 29.4)	28.2 (27.6, 28.9)	0.398
Sex				0.122
Male	374 (62.8%)	303 (64.3%)	71 (56.8%)	
Female	222 (37.2%)	168 (35.7%)	54 (43.2%)	
Apgar Score 1 min	8 (7, 8)	8 (7, 8)	8 (7, 8)	0.080
Apgar Score 5 min	9 (8, 9)	9 (9, 9)	9 (8, 9)	0.137
Antenatal steroids (%)	571 (95.8%)	454 (96.4%)	117 (93.6%)	0.166
Prolonged rupture of membrane (%)	93 (15.6%)	75 (15.9%)	18 (14.4%)	0.676
Pregnancy-induced hypertension (%)	76 (12.8%)	55 (11.7%)	21 (16.8%)	0.127
Gestational diabetes (%)	77 (12.9%)	65 (13.8%)	12 (9.6%)	0.213
Histologic chorioamnionitis (%)	68 (11.4%)	48 (10.2%)	20 (16.0%)	0. 069

Median with interquartile range (IQR; P25–P75) for continuous variables and % for categorical variables. *p* < 0.05.

Comparative analyses revealed significant intergroup differences (*p* < 0.05) in arterial pH, blood glucose, white blood cell (WBC) counts, platelet counts (PLT), oxygenation index (OI), prevalence of septic shock, and exposure to methylprednisolone or epinephrine. Non-survivors had significantly higher Gram-negative bacteremia prevalence (64.8% vs. 39.7%; *p* < 0.001) but lower Gram-positive bacteremia incidence (22.4% vs. 41.0%; *p* < 0.001). Gestational age, absolute neutrophil counts, procalcitonin (PCT), and C–reactive protein (CRP) were comparable between groups (Table 2).

**Table 2:** Clinical and laboratory characteristics of VLBW infants with LOS.

Variables	Total (*n* = 596)	Survivor (*n* = 471)	Non-survivor (*n* = 125)	*p*
pH	7.345 (7.276, 7.407)	7.354 (7.298, 7.410)	7.268 (7.142, 7.377)	**<0.001**
Blood lactate (mmol/L)	1.4 (0.9, 3.1)	1.2 (0.8, 2.0)	4.2 (2.5, 4.1)	**<0.001**
Blood glucose (mmol/L)	6.6 (5.5, 8.1)	6.5 (5.2, 7.9)	7.1 (6.0, 8.5)	**<0.001**
White blood cell counts (10^9/L)	7.5 (4.4, 12.2)	8.0 (4.5, 12.4)	5.6 (3.7, 10.0)	**0.003**
Absolute neutrophil counts (10^9/L)	3.3 (1.5, 6.8)	3.6 (1.5, 7.3)	2.6 (1.5, 5.4)	0.093
Platelet counts (10^9/L)	133 (76, 219)	142 (86, 228)	102 (49, 161)	**<0.001**
Procalcitonin (PCT, ng/ml)	3.8 (0.4, 15.0)	3.6 (0.4, 14.0)	4.3 (0.5, 15.3)	0.142
C-reactive protein (CRP, mg/L)	13.0 (3.0, 35.0)	12.0 (2.6, 34.5)	15.0 (4.0, 36.0)	0.148
Oxygen index (OI)	5.7 (1.9, 10.9)	4.2 (1.5, 8.0)	14.9 (10.8, 21.4)	**<0.001**
Sepcies of pathogens of blood culture				
Gram-positive bacteria	221 (37.1%)	193 (41.0%)	28 (22.4%)	**<0.001** ^†^
Gram-negative bacteria	268 (45.0%)	187 (39.7%)	81 (64.8%)	**<0.001** ^†^
Fungemia	18 (3.0%)	16 (3.4%)	2 (1.6%)	0.390
Sepsis shock				**<0.001**
No	164 (27.5%)	154 (32.7%)	10 (8.0%)	
Yes	432 (72.5%)	317 (67.3%)	115 (92.0%)	
Methylprednisolone use				**<0.001**
No	522 (87.6%)	447 (94.9%)	75 (60.0%)	
Yes	74 (12.4%)	24 (5.1%)	50 (40.0%)	
Epinephrine use				**<0.001**
No	514 (86.2%)	435 (92.4%)	79 (63.2%)	
Yes	82 (13.8%)	36 (7.6%)	46 (36.8%)	
Oxygen Index Grade				**<0.001**
OI < 4	232 (38.9%)	230 (48.8%)	2 (1.6%)	
4 ≤ OI < 8	129 (21.6%)	119 (25.3%)	10 (8.0%)	
8 ≤ OI	235 (39.5%)	122 (25.9%)	113 (90.4%)	

Median with interquartile range (IQR; P25–P75) for continuous variables and % for categorical variables. ^†^Group comparisons were performed with chi-square tests followed by Bonferroni-correction for multiple testing. *p* < 0.05.

### 3.2 Association between blood lactate and Sepsis–related mortality

In unadjusted analysis, each 1 mmol/L increment in blood lactate conferred a 90% elevation in sepsis–related mortality risk (OR = 1.90, 95%CI: 1.68–2.14; *p* < 0.001). Sequential adjustment for demographic and therapeutic confounders (Model 2: birth weight, gestational age, sex, septic shock, methylprednisolone use, and epinephrine use minimally attenuated the association [adjusted odds ratio (17), aOR = 1.87, 95% CI: 1.62–2.16; *p* < 0.001]. Full adjustment in Model 3 incorporated blood culture results, tachycardia (>180 bpm), bradycardia (<85 bpm), pH, glucose, white blood cell counts, absolute neutrophil counts, platelet counts (PLT), procalcitonin (PCT), C-reactive protein (CRP), and OI). This comprehensive adjustment significantly strengthened the association (aOR = 1.96, 95% CI: 1.56–2.45; *p* < 0.001; Table 3).

**Table 3:** Association between blood lactate levels and sepsis-related mortality in very low birth weight infants with late-onset sepsis: multivariable logistic regression analysis.

Variables	Model 1		Model 2		Model 3	
	OR (95% CI)	*p*	OR (95% CI)	*p*	OR (95% CI)	*p*
Lactate (mmol/L)	1.90 (1.68–2.14)	**<0.0001**	1.87 (1.62–2.16)	**<0.0001**	1.96 (1.56–2.45)	**<0.0001**

Results from sequential logistic regression models estimating the association between blood lactate levels (per 1 mmol/L increase) and sepsis-related mortality. Model 1: unadjusted; Model 2: adjusted for Birth weight, Gestational age, Sex, Sepsis shock, Methylprednisolone use, and Epinephrine use; Model 3: fully adjusted for Birth weight, Gestational age, Sex, Sepcies of pathogens bacteria, Sepsis shock, Methylprednisolone use, Epinephrine use, tachycardia (>180 bpm), bradycardia (<85 bpm), pH, Glucose, white blood cell counts, absolute neutrophil counts, platelet counts (PLT), procalcitonin (PCT), C-reactive protein (CRP), and oxygenation index (OI). OR, odds ratio; CI, confidence interval. *p* < 0.05.

### 3.3 Nonlinear threshold dynamics

**Figure 2:**
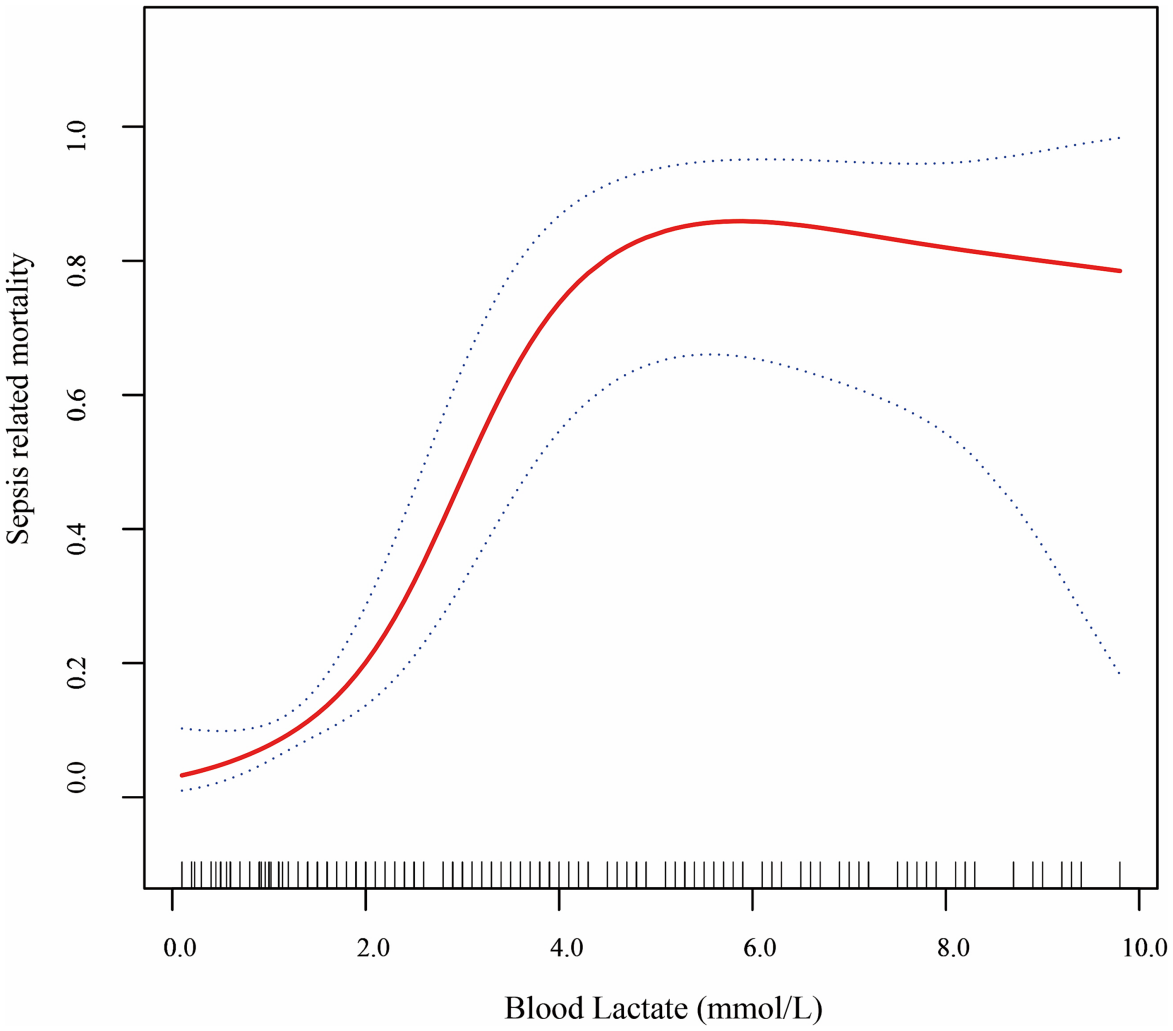
Smooth fitting curves demonstrated a non-linear relationship between lactate level and sepsis-related mortality in VLBW infants with LOS.

The association between blood lactate levels and sepsis–related mortality demonstrated a nonlinear pattern (Figure 2). Piecewise regression analysis identified two critical lactate thresholds at 2.2 mmol/L (95% CI: 1.9–2.5) and 4.0 mmol/L (95% CI: 3.7–4.3), defining a triphasic mortality risk relationship that significantly outperformed linear models (likelihood test, *p* < 0.001; Table 4). Concentrations below 2.2 mmol/L demonstrated no significant mortality association (aOR = 1.84, 95% CI: 0.64–5.34; *p* = 0.260). Within the critical 2.2–4.0 mmol/L transition zone, each mmol/L increment conferred a 7.0-fold mortality risk elevation (aOR = 7.00, 95% CI: 2.13–22.8; *p* < 0.001), representing the steepest risk gradient observed. Beyond 4.0 mmol/L, the association attenuated to non-significance (aOR = 0.90; 95% CI: 0.52–1.43; *p* = 0.568). Finally, bootstrap validation with 1,000 replicates confirmed robust threshold stability.

**Table 4:** threshold effects analysis between blood lactate level and sepsis-related mortality in VLBW infants.

Outcome	Adjusted OR (95% CI)	*p*-value
Model 3: Fitting model by standard linear regression	1.96 (1.56–2.45)	**<0.001**
Model 4: Fitting model by piecewise linear regression		
Inflection point	2.2 mmol/l, 4.0 mmol/L	
< 2.2 mmol/L	1.84 (0.64–5.34)	0.260
2.2 mmol/L to 4.0 mmol/L	7.00 (2.13–22.78)	**<0.001**
≥ 4.0 mmol/L	0.90 (0.52–1.43)	0.568
*p-value* for likelihood test		**<0.001**

Birth weight, Gestational age, Sex, Sepsis shock, tachycardia (>180 bpm), bradycardia (<85 bpm), Methylprednisolone use, Epinephrine use, Sepcies of pathogens bacteria, pH, Glucose, white blood cell counts, absolute neutrophil counts, platelet counts (PLT), procalcitonin (PCT), C-reactive protein (CRP), and oxygenation index (OI) were adjusted in the threshold effect analysis. *p* < 0.05.

### 3.4 Stratified analysis

Stratified analyses confirmed consistent lactate-mortality associations across demographic and clinical subgroups (Supplementary Figure 1; Supplementary Table 1), with nonsignificant effect modification for: (1) Septic shock (present aOR = 1.91, 95% CI: 1.63–2.24; absent aOR = 1.42, 95% CI: 0.96–2.11; *P*_interaction_ = 0.502); (2) Methylprednisolone (non-users aOR = 1.90, 95% CI: 1.62–2.23; users aOR = 1.56, 95% CI: 0.95–2.58; *P*_interaction_ = 0.376). Notable heterogeneity emerged in two high–risk subgroup: the mortality risk per 1 mmol/L lactate increase was 91% higher in epinephrine-treated infants (aOR = 3.40, 95% CI: 1.40–8.27; *p* = 0.007) versus non-recipients (aOR = 1.78, 95% CI: 1.53–2.08; *p* < 0.001). Although the interaction term indicated differential effects (*P*_interaction_ = 0.094), this did not achieve statistical significance after Bonferroni correction for multiple comparisons (corrected *α* = 0.01).

Stratified analyses confirmed significant effect measure modification of the lactate-mortality association by respiratory failure severity (*P*_interaction_ = 0.180). Neonates with minimal hypoxemia (OI < 4) demonstrated no significant mortality association with initial lactate levels. Conversely, in moderate hypoxemia (4 ≤ OI < 8), each 1 mmol/L lactate increment conferred a 2.61-fold higher mortality risk (aOR = 2.61, 95%CI: 1.38–4.94; *p* = 0.003). Notably, the association attenuated in severe hypoxemia (OI ≥ 8), with a 1.81-fold mortality increase per 1 mmol/L elevation (aOR = 1.81, 95%CI: 1.35–2.44; *p* < 0.001).

## 4 Discussion

This study confirms, in alignment with recent seminal sepsis researches, that blood lactate elevation at the onset of sepsis were associated with mortality across different populations (4, 18, 19). However, our findings expose fundamental developmental divergences in threshold dynamics and pathophysiological interpretations. While pediatric and adult studies consistently report linear or single-threshold models [e.g., lactate >2.0 mmol/L uniformly increasing mortality risk (4, 19, 20)], we demonstrate a triphasic, nonlinear risk architecture unique to VLBW infants: (1) a neutral zone less than 2.2 mmol/L, (2) a sharply escalating hazard phase between 2.2 to 4.0 mmol/L, and (3) an attenuated phase when beyond 4.0 mmol/L. In adult sepsis, persistent hyperlactatemia exceeding 4.0 mmol/L consistently correlates with increased mortality risk (21). Conversely, in preterm infants, the association between hyperlactatemia above this threshold and mortality is markedly attenuated. This pathophysiological divergence primarily reflects the neonate’s immature organ systems and heightened mitochondrial vulnerability, which predispose them to accelerated progression towards irreversible cellular injury once a critical point is reached (22). Consequently, these findings highlight a critically narrow therapeutic window during the early stages of sepsis in VLBW infants, underscoring the imperative for prompt intervention to prevent irreversible deterioration.

Exploratory Subgroup Findings Under Linearity Assumption:

Our primary analysis identified critical lactate thresholds at 2.2 and 4.0 mmol/L. Prespecified subgroup analyses revealed contextual heterogeneity in mortality risk associations (Supplementary Table 1; Supplementary Figure 1), with epinephrine exposure demonstrating the most significant pattern: each 1 mmol/L lactate increase was associated with different mortality risk in non-survivors (aOR = 3.40, 95% CI: 1.40–2.87) versus survivors (aOR = 1.78, 95% CI: 1.53–2.08), representing near 2-fold risk amplification. We explicitly frame this as exploratory given the non-significant interaction (*P*_interaction_ = 0.094) and its absence in adult cohorts.

Mechanistically, hyperlactatemia during epinephrine infusion signals developmental vulnerability unique to neonates. In epinephrine-dependent shock, hyperlactatemia reflects exhaustion of physiological compensation—characterized by severe mitochondrial respiratory chain dysfunction and impaired oxidative phosphorylation (22). The developmentally immature preterm infant exhibits progressive metabolic failure due to constrained organ reserve capacity and impaired mitochondrial function. Consequently, the increasing need for epinephrine support was a critical factor associated with the risk of mortality among VLBW infants with LOS (10, 23, 24). While parallels exist with adult sepsis—where lactate >2 mmol/L predicts >40% mortality (25)—our findings underscore critical developmental distinctions: neonates demonstrate heightened metabolic vulnerability at lower lactate thresholds due to immature mitochondrial function; epinephrine uniquely amplifies lactate-associated mortality risk in this population through catecholamine-driven suppression of hepatic lactate clearance; and preterm mitochondrial immaturity fundamentally modifies this relationship by constraining respiratory chain reserve capacity. While these insights support lactate’s potential utility as both a biomarker of metabolic stress and a clinical decision aid, we emphasize that the observed epinephrine-lactate interaction remains exploratory; consequently, application of this specific risk relationship to guide clinical thresholds requires prospective validation before implementation. Future research must prioritize real-time mitochondrial function assessment (e.g., cytochrome c oxidase activity monitoring) during catecholamine infusion to establish developmentally appropriate intervention thresholds that account for gestational-age-specific vulnerabilities.

Simultaneously, hypoxemia severity demonstrated potential modification of lactate-mortality associations (*P*_interaction_ = 0.180)—mechanistically linked through hypoxia-driven cellular energy failure—necessitating stratified analyses to elucidate oxygenation-dependent risk stratification patterns. First, in moderate hypoxemia (4 ≤ OI < 8), each 1 mmol/L lactate increase conferred a 2.61-fold adjusted mortality risk (aOR = 2.61, 95%CI: 1.38–4.94). The biological plausibility stems from synergistic pathophysiological mechanisms: tissue hypoxia accelerates anaerobic metabolism while impaired hepatic clearance coincides with hypoxia-inducible factor (HIF-1α)-mediated inflammatory cascades that exacerbate lactate accumulation (26, 27). Consequently, blood lactate may serve as a barometer of unmet metabolic demand, where interventions like lung–protective ventilation may reverse the trajectory, indicating this phase may represent a critical therapeutic window to prevent irreversible clinical deterioration (8). Notably, the attenuated association of lactate with mortality in severe to refractory hypoxemia subgroups (OI ≥ 8, aOR = 1.81, 95%CI: 1.49–2.20) highlights its predictive limitations. In this terminal phase, lactate transitions from a hypoxia–driven prognostic marker to an epiphenomenal biomarker of global metabolic derangement, may reflect fundamental shifts in lactate pathophysiology during severe respiratory failure–severe hypoxia induces mitochondrial electron transport chain dysfunction, leading lactate levels decouple from tissue perfusion status in terminal respiratory failure (22). Moreover, sepsis related mortality risk is more likely dominated by irreversible multiorgan dysfunction (e.g., cardiogenic shock, sepsis–induced immunometabolism collapse), where lactate elevation reflects systemic bioenergetic failure rather than isolated hypoxic injury (8, 28–30). This biological context explains why advanced respiratory support modalities (e.g., ECMO) that improve oxygenation without reversing cellular metabolic dysfunction may further attenuate lactate-mortality associations (31).

Furthermore, studies stratifying the lactate-mortality relationship by oxygenation index (OI) remain scarce in adult and pediatric sepsis cohorts. This knowledge gap primarily stems from the unique developmental physiology of VLBW infants—manifested through surfactant deficiency impairing pulmonary compliance, immature myocardial contractility limiting cardiac output reserve, and attenuated hypoxic ventilatory response blunting respiratory compensation—whereby sepsis invariably triggers rapid respiratory decompensation, significantly increasing dependence on respiratory support (10, 16). Moreover, accurate OI determination requires serial arterial blood gas analyses—an invasive procedure with non-negligible complications. Concurrent lactate-OI measurement further demands precise temporal synchronization, creating operational barriers that restrict dataset completeness in real-world settings. Although in adult septic shock cohort the oxygen delivery and oxygen uptake cannot be used as prognostic indicators, lactate elevation consistently correlates with mortality (32).

Building on these insights, our analysis reframes the determinants of neonatal mortality beyond simplistic time-to-treatment metrics, revealing three interdependent pathways: therapeutic precision, systemic circadian vulnerability, and terminal metabolic collapse. First, although empiric antimicrobial resistance demonstrated a threefold elevation in crude mortality risk (10.4% vs. 3.2%, *p* < 0.001) (Supplementary Table 2), its transition to borderline non-significance following multivariable adjustment (aOR = 2.29, 95%CI: 0.47–11.08; *p* = 0.302) (Supplementary Table 3) indicates the critical role of hyperlactatemia-driven multiorgan dysfunction. This aligns with the physiological cascade whereby ineffective antimicrobial control permits unchecked infection progression, driving tissue hypoperfusion and lactic acidosis—this pathological manifestation objectively quantified by lactate elevation. Crucially, the comparable time-to-antibiotics across groups (92.0% vs. 91.7%, *p* = 0.913; Supplementary Table 2) demonstrates that appropriate antimicrobial selection is paramount, outweighing expedited administration alone (33).

Second, the trend toward higher mortality with night-onset symptoms (00:00 am–08:00 am; aOR = 2.01, 95% CI: 0.78–5.17; *p* = 0.147; Supplementary Table 3) signals potential circadian vulnerabilities in care delivery. Though statistically non-significant—possibly due to sample size limitations—the effect size warrants attention given established evidence of off-hour reductions in NICU staffing acuity and diagnostic vigilance (34). Such systemic constraints may narrow the effective therapeutic window through delayed recognition or suboptimal clinical decision-making, although further investigation is needed (35).

Notably, serum lactate emerged as the predominant independent predictor (aOR = 2.0 per mmol/L, 95% CI: 1.60–2.55; *p* < 0.001; Supplementary Table 3), surpassing the implications of both antimicrobial resistance and circadian effects. This biomarker consolidates multifactorial insults (pathogen virulence, therapeutic inadequacy, or delayed response) into a final common pathophysiological state of disease—cellular energy failure. Our findings align with current research affirming the relationship between lactate levels and mortality in neonates with LOS, establishing it as the most proximal indicator of irreversible metabolic collapse.

Leveraging this insight into lactate’s prognostic significance, we propose extending its application to earlier stages of neonatal care. Specifically, we hypothesize that umbilical cord blood lactate monitoring could serve as an early predictor of sepsis-associated mortality in neonates. To evaluate this hypothesis against existing evidence, although umbilical arterial hyperlactatemia (> 7.0 mmol/L) is significantly associated with sepsis incidence (aHR = 2.13; 95% CI 1.85–2.45; *p* < 0.001), this relationship does not extend to mortality (36), nor does it predict early-onset sepsis (37). Notably, venous lactate >3.38 mmol/L at 6 h postnatally emerges as a potential sepsis predictor (sensitivity 57.9%; specificity 68.5%; *p* = 0.032) (37). This temporal divergence gains support from a multicenter cohort of 2,499 preterm infants (<29 weeks), where day-1 lactate elevation independently predicted intraventricular hemorrhage (aOR = 1.18; 95% CI 1.03–1.37; *p* = 0.005) and bronchopulmonary dysplasia (aOR = 1.23; 95% CI 1.06–1.43; *p* = 0.005), yet has limit association with sepsis or mortality (all *p* ≥ 0.05) (38). Consistent with this pattern, serum lactate within the first postnatal hour in 60 VLBW infants lacked prognostic value for sepsis/mortality (all *p* > 0.05) despite correlating with retinopathy of prematurity (*p* = 0.042) and bronchopulmonary dysplasia (*p* = 0.015) (39). Collectively, these findings establish cord blood lactate as a primary biomarker of intrauterine hypoxic insults rather than sepsis-related pathology. Consequently, while umbilical lactate maintains value in evaluating perinatal compromise, existing evidence underscores its restricted utility for early risk stratification of sepsis-associated mortality, thereby warranting the pursuit of dynamic postnatal biomarker frameworks.

Current research on lactate clearance in sepsis predominantly draws from human studies in pediatric and adult populations, establishing it as a potential marker of treatment response and prognosis. For instance, in adult severe sepsis with organ dysfunction, early higher lactate clearance linked to decreased mortality rate (40). Similarly, in febrile African children, 8-h clearance independently predicted 72-h survival (41). These findings position lactate clearance as a dynamic indicator of resolving hypoperfusion, offering potential advantages over static measurements in monitoring resuscitation efficacy. However, discrepancies among studies challenge the superiority of clearance over baseline lactate levels. In Sepsis-3-defined adult septic shock, both metrics predicted mortality (OR: 1.27, 95% CI: 1.21–1.34 for lactate levels; OR: 0.992, 95% CI: 0.989–0.995 for lactate clearance), yet baseline levels demonstrated better discriminatory power [area under the curve (AUC) 0.70 vs. 0.65; *p* < 0.01], with thresholds like ≥2 mmol/L providing high sensitivity (85.3%) (42). Clearance alone did not independently associate with outcomes in some analyses (aRR: 0.75, 95% CI: 0.38–1.50) (43), highlighting inconsistencies that may stem from heterogeneous study designs, varying thresholds, or population differences. Such divergences underscore the need for cautious interpretation, as clearance’s prognostic edge is not universal and may be context-dependent.

Mechanistically, lactate clearance reflects restored aerobic metabolism amid hypoperfusion, anaerobic glycolysis, or adrenergic stress, signaling reversal of tissue hypoxia. However, multifactorial influences complicate its specificity, including microcirculatory defects, age-related pathophysiology, and non-septic confounders like drug-induced elevations (e.g., steroids) (44). In sepsis, elevated lactate overwhelmingly marks illness severity, but clearance may not fully isolate perfusion recovery from these overlapping processes, potentially leading to overestimation of its standalone value (44).

In neonates, these mechanisms manifest uniquely due to immature hepatic and renal function, heightened vulnerability to shock, and comorbidities like prematurity, limiting direct extrapolation from other cohorts. Neonatal sepsis mortality often results from unrecognized hypoperfusion, where lactate clearance could theoretically guide interventions (43). Yet, age-dependent lactate dynamics—such as slower clearance rates—necessitate tailored thresholds, as generic metrics may overlook developmental immaturities (41, 43). Clinically, this suggests clearance as a complementary NICU tool for treatment monitoring, potentially enabling fluid resuscitation to address mortality in preterm late-onset sepsis. Nonetheless, sparse neonate-specific data risk misinterpretation without validation. To resolve these gaps, large-scale prospective studies in neonatal cohorts are essential, incorporating serial measurements and time-series analyses (e.g., delta metrics or AUC) against endpoints like mortality, while controlling for confounders. Multicenter designs stratified by gestational age could refine protocols, bridging evidence from non-neonatal studies to precise neonatal applications (44).

This study confirms lactate’s consistent mortality association in sepsis across different age groups, but reveals a specific physiologic pattern among VLBW infants. Unlike the linear risk between blood lactate and sepsis mortality in adults and children—VLBW infants demonstrate rapid risk acceleration within a narrow mid-range lactate band (2.2 mmol/L—4.0 mmol/L), followed by unexpected prognostic dissociation at higher values. This reflects preterm biology’s constraints: underdeveloped organs and fragile mitochondria limit compensatory reserve, accelerating irreversible damage once lactate surpasses critical levels—contrasting adults’ sustained high-lactate mortality risk (45). Clinically, these findings highlight the need for population-specific protocols: in preterm infants, prioritize targeted interventions for those with lactate levels of 2.2–4.0 mmol/L as a potential intervention window. This customized approach minimizes the risks of applying adult guidelines to neonates, promoting prompt, individualized interventions in this vulnerable group to optimize outcomes.

## 5 Limitation

The principal strength of this study is the identification of lactate thresholds that enable clinically actionable risk stratification in VLBW infants with late-onset sepsis. Several limitations, however, warrant acknowledgment. Notably, serum lactate was assessed as a single static measurement at sepsis onset, whereas serial monitoring might provide enhanced prognostic value; moreover, the single-center retrospective design introduces risks of selection bias and residual confounding, compounded by the exclusion of 11 infants owing to treatment withdrawal—a real-world limitation in neonatal care that may underestimate mortality rates and underrepresent high-risk subgroups, such as those with severe hyperlactatemia or epinephrine dependence. Additionally, conducted in Suzhou, China, our findings may lack generalizability to diverse global NICU populations, where variations in genetic factors, pathogen distributions, and resource availability could alter outcomes. These validated thresholds outline a critical transition phase (2.2–4.0 mmol/L) that may represent an optimal window for targeted metabolic resuscitation in septic VLBW infants, although observational data preclude definitive claims of improved survival. Consequently, to address this evidence gap and enhance broader applicability, we recommend validation in multicenter, international prospective cohorts that incorporate intention-to-treat analyses or sensitivity assessments, alongside a randomized controlled trial (RCT) to rigorously evaluate whether lactate-guided interventions enhance outcomes compared to standard care; such efforts would provide causal insights, confirm subgroup interactions (e.g., epinephrine effects), and support the incorporation of these thresholds into evidence-based neonatal sepsis protocols, ultimately refining lactate’s utility as a biomarker.

## 6 Conclusion

In conclusion, our research identified critical serum lactate thresholds (2.2 mmol/L and 4.0 mmol/L) in VLBW infants with LOS, revealing a non-linear association with sepsis-related mortality and offering a quantifiable biomarker for risk stratification. Although these findings highlight a potential intervention window within the 2.2–4.0 mmol/L range, the observational design limits causal inferences regarding improved outcomes. Future research should prioritize dynamic lactate monitoring, multimodal assessments (e.g., near-infrared spectroscopy), and randomized controlled trials to evaluate lactate-guided resuscitation strategies, thereby refining evidence-based protocols for neonatal sepsis management.

## Data Availability

The datasets presented in this study can be found in online repositories. The names of the repository/repositories and accession number(s) can be found at: https://data.mendeley.com/preview/zf89djy79t.

## Footnotes

^1^http://www.empowerstats.com/cn/, X&Y solutions, Inc., Boston, MA
