# Supplementary material for: Phase-specific mortality risk of serum lactate thresholds in very low birth weight infants with late-onset sepsis: a retrospective cohort study
Source: Front Med (Lausanne). 2025 Sep 4;12:1662406. doi: 10.3389/fmed.2025.1662406 (PMC12443540; doi:10.3389/fmed.2025.1662406)
Supplement: Supplementary file 1 [file Data_Sheet_1.PDF]

Supplementary Table S1: Stratified Analysis of Lactate and Sepsis-Related Mortality

Variables	n (%)	aOR (95%CI)	*P*	*P* interaction
**All patients**	596 (100.00%)	1.90 (1.64 ~ 2.19)	**<.001**	
**Sepsis Shock**				0.502
no	164 (27.52%)	1.42 (0.96 ~ 2.11)	0.081	
yes	432 (72.48%)	1.91 (1.63 ~ 2.24)	**<.001**	
**Methylprednisolone use**				0.376
no	522 (87.58%)	1.90 (1.62 ~ 2.23)	**<.001**	
yes	74 (12.42%)	1.56 (0.95 ~ 2.58)	**0.081**	
**Epinephrine use**				0.094
no	514 (86.24%)	1.78 (1.53 ~ 2.08)	**<.001**	
yes	82 (13.76%)	3.40 (1.40 ~ 8.27)	**0.007**	
**Oxygenation Index**				0.180
OI < 4	232 (38.93%)	0.00 (0.00 ~ 7.82)	0.185	
4 ≤ OI < 8	129 (21.64%)	2.61 (1.38 ~ 4.94)	**0.003**	
OI ≤ 8	235 (39.43%)	1.81 (1.49 ~ 2.20)	**<.001**	

Note:

1. All subgroup analyses followed prespecified protocols.
2. Interaction effects tested using multiplicative terms in multivariable logistic regression, adjusted for birthweight, sepcies of pathogens of blood culture, tachycardia (>180bpm), bradycardia (<85bpm), white blood cell counts, platelet counts, procalcitonin, and C-reactive protein.
3. Oxygenation Index strata were defined as OI < 4, 4 ≤ OI < 8 (mild), and OI ≥ 8 (moderate to severe) to ensure adequate statistical power.
4. Interaction *P*-values significant if < 0.01 (Bonferroni correction).
5. Abbreviations: OR, odds ratio; CI, confidence interval; OI, oxygenation index.

Supplementary Table 2. Demographics of VLBW infants with late-onset sepsis episodes and characteristics of empiric antimicrobials.

Variables	Survivor (n = 471)	Non-survivor (n = 125)	*P*-value
**Time to first antibiotics less than three hours**	432 (91.7%)	115 (92.0%)	0.913
**Empiric Therapy Concordance with Blood Culture Profiles**			
Susceptible to Antimicrobial Agents	386 (82.0%)	98 (78.4%)	0.366
Resistant to Antimicrobial Agents	15 (3.2%)	13 (10.4%)	**<0.001**
Indeterminate Therapeutic Efficacy Attributable to Blood Culture-Negative Status	70 (14.8%)	14 (11.2%)	0.296
**Initial sepsis symptom onset during off-hours**	47 (9.9%)	19 (15.2%)	0.098
**Initial sepsis symptom onset between Nighttime (00:00 am–08:00 am)**	91 (19.3%)	30 (24.0%)	0.248

Note: Categorical variables are reported as number and percentage.

Supplementary Table 3. Association Between empiric antimicrobials and Sepsis-Related Mortality in Very Low Birth Weight Infants with Late-Onset Sepsis: Multivariable Logistic Regression Analysis

Variables	Odd ratio	Std.err	95% CI	*P*-value
**Time to first antibiotics less than three hours**	1.660	0.727	0.703 – 3.918	0.247
**Empiric Therapy Concordance with Blood Culture Profiles**	2.291	1.842	0.474 – 11.077	0.302
**Initial sepsis symptom onset during off-hours**	0.274	0.196	0.067 – 1.111	0.070
**Initial sepsis symptom onset between Nighttime (00:00–08:00)**	2.011	0.969	0.782 – 5.172	0.147
**Lactate (mmol/L)**	2.020	0.241	1.598 – 2.553	**<0.000**

Note: Fully adjusted for Birth weight, Gestational age, Sex, Sepcies of pathogens bacteria, Sepsis shock, Methylprednisolone use, Epinephrine use, tachycardia (>180 bpm), bradycardia (<85 bpm), pH, Glucose,white blood cell counts, absolute neutrophil counts, platelet counts (PLT), procalcitonin (PCT), C-reactive protein (CRP), **Time to first antibiotics less than three hours**, **Efficacy to Pathogens of Initial Empirical Antibiotics, Initial sepsis symptom onset during off-hours, Initial sepsis symptom onset between Nighttime (00:00 am–08:00 am),** and oxygenation index (OI). OR: odds ratio; CI: confidence interval.

Supplementary Figure 1. Mortality Risk Association of Serum Lactate Stratified by Prespecified Clinical Variables

**Variables**	**n (%)**	**aOR (95%CI)**	***P***	***P* interaction**
**All patients**	596 (100.00)	1.90 (1.64 ~ 2.19)	<.001	
**Sepsis Shock**				0.502
no	164 (27.52%)	1.42 (0.96 ~ 2.11)	0.081	
yes	432 (72.48%)	1.91 (1.63 ~ 2.24)	<.001	
**Methylprednisolone use**				0.376
no	522 (87.58%)	1.90 (1.62 ~ 2.23)	<.001	
yes	74 (12.42%)	1.56 (0.95 ~ 2.58)	0.081	
**Epinephrine use**				0.094
no	514 (86.24%)	1.78 (1.53 ~ 2.08)	<.001	
yes	82 (13.76%)	3.40 (1.40 ~ 8.27)	0.007	
**Oxygenation Index**				0.180
OI < 4	232 (38.93%)	0.00 (0.00 ~ 7.82)	0.185	
4 ≤ OI < 8	129 (21.64%)	2.61 (1.38 ~ 4.94)	0.003	
OI ≤ 8	235 (39.43%)	1.81 (1.49 ~ 2.20)	<.001	

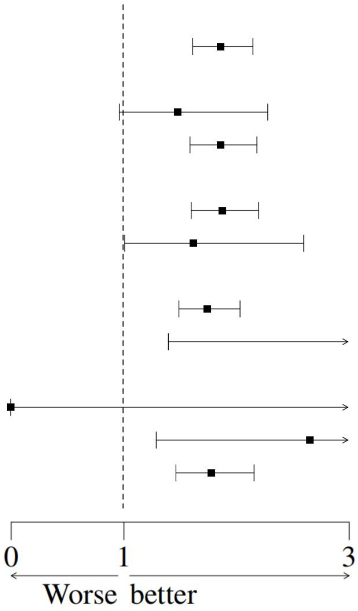

Forest plot of adjusted odds ratios (aOR) for mortality per 1-mmol/L serum lactate increase.
